# Longitudinal Changes in Body Composition, Resting Metabolic Rate, and Inflammation After Sleeve Gastrectomy: Associations with Dietary Polyunsaturated Fatty Acid Balance

**DOI:** 10.3390/metabo16090664

**Published:** 2026-09-09

**Authors:** Ghazal Mohammad, Abeer S. Alzaben, Yazeed Alshuweishi, Sarah F. Faludah, Meshal Al-Sharafa, Shaima Alothman, Arwa S. Altalhi, Doaa S. Aljasser, Dalal F. ALShamri, Saeed Al Shlwi, Abdullah Aldriee, Latifah Alanzi, Fayzah S. Alotaibi, Hanaa A. Yamani, Ali A. Alshatwi, Alaa A. Al-Masud

**Affiliations:** 1 Nourah’s Tissue Biobank, Natural and Health Science Research Center, Princess Nourah bint Abdulrahman University, P.O. Box 84428, Riyadh 11671, Saudi Arabia; 443203605@student.ksu.edu.sa (G.M.); sffaloudah@pnu.edu.sa (S.F.F.); mmalsharafa@pnu.edu.sa (M.A.-S.); 2 Department of Food Science and Nutrition, College of Food and Agriculture Sciences, King Saud University, P.O. Box 2460, Riyadh 11451, Saudi Arabia; alshatwi@ksu.edu.sa; 3 Department of Health Sciences, College of Health and Rehabilitation Sciences, Princess Nourah bint Abdulrahman University, Riyadh 11671, Saudi Arabia; asalzaben@pnu.edu.sa; 4 Department of Clinical Laboratory Sciences, College of Applied Medical Sciences, King Saud University, Riyadh 12372, Saudi Arabia; yalshuweishi@ksu.edu.sa; 5 Centre of Excellence in Biotechnology Research (CEBR), King Saud University, Riyadh 11451, Saudi Arabia; 6 Lifestyle and Health Research, Natural and Health Sciences Research Center, Princess Nourah bint Abdulrahman University, P.O. Box 84428, Riyadh 11671, Saudi Arabia; shaalothman@pnu.edu.sa (S.A.); arsaltalhi@pnu.edu.sa (A.S.A.); 7 Epidemiology and Biostatistics Section, Health Sciences Research Center, Princess Nourah bint Abdulrahman University, Riyadh 11671, Saudi Arabia; 8 Department of Nutrition, King Abdullah bin Abdulaziz University Hospital, Princess Nourah bint Abdulrahman University, Riyadh 11671, Saudi Arabia; dfalshamri@kaauh.edu.sa; 9 Department of Surgery, King Abdullah bin Abdulaziz University Hospital, Princess Nourah bint Abdulrahman University, Riyadh 11671, Saudi Arabia; ssalshlwi@kaauh.edu.sa; 10 Department of Pathology and Laboratory Medicine, King Abdullah bin Abdulaziz University Hospital, Princess Nourah bint Abdulrahman University, Riyadh 11671, Saudi Arabia; aaaldriee@kaauh.edu.sa (A.A.); lmalanzi@kaauh.edu.sa (L.A.); fsalotaibi@kaauh.edu.sa (F.S.A.); 11 Department of Biology, College of Science, University of Jeddah, Jeddah 23881, Saudi Arabia; hayamani@uj.edu.sa; 12 Department of Biology, Faculty of Science, Princess Nourah bint Abdul Rahman University, Riyadh 11671, Saudi Arabia

**Keywords:** sleeve gastrectomy, omega-6 fatty acids, omega-3 fatty acids, inflammation, body composition

## Abstract

**Highlights:**

**What are the main findings?**

- Sleeve gastrectomy was associated with distinct postoperative trajectories, including progressive reductions in body weight, adiposity, and CRP, alongside an early decline and subsequent stabilization of resting metabolic rate.
- Dietary omega-6/omega-3 ratio was not significantly associated with body-composition, metabolic, or inflammatory outcomes, while individual dietary PUFA measures showed limited exploratory associations.

**What are the implications of the main findings?**

- Postoperative metabolic adaptation is not uniform across physiological domains, highlighting the value of longitudinal monitoring of body composition, energy metabolism, and inflammation after sleeve gastrectomy.
- The limited associations with dietary PUFA measures suggest that postoperative changes are predominantly related to surgery-associated temporal adaptations, while the role of dietary fatty acid balance requires confirmation in larger, adequately powered studies.

**Abstract:**

Background/Objectives: Sleeve gastrectomy induces substantial changes in body composition, energy metabolism, and systemic inflammation, but their temporal patterns during early postoperative follow-up remain incompletely characterized. This pilot study primarily characterized longitudinal changes in body composition, resting energy metabolism, and inflammatory biomarkers during the first nine months following sleeve gastrectomy. A secondary exploratory objective examined associations between the dietary omega-6/omega-3 ratio and these outcomes. Methods: Thirty-seven adults (67.6% female; mean age 35.1 ± 11.3 years) undergoing sleeve gastrectomy were prospectively assessed at baseline and 3, 6, and 9 months postoperatively. Dietary intake was assessed using three-day food records. Body composition, resting metabolic rate (RMR), respiratory quotient, and inflammatory biomarkers (CRP, IL-6, and IL-10) were evaluated longitudinally. Linear mixed models adjusted for age, sex, and comorbidity were used to assess temporal changes and dietary associations. Results: Distinct postoperative trajectories were observed across outcomes. BMI declined progressively from 43.76 ± 6.44 to 28.49 ± 3.77 kg/m^2^ and total body fat from 52.32 ± 5.76% to 39.62 ± 5.71% (both *p* < 0.001 for time). In contrast, RMR showed a marked early decline by 3 months, followed by relative stabilization (*p* < 0.001). CRP progressively decreased from 12.84 ± 12.60 to 2.96 ± 2.95 mg/L (*p* = 0.001), whereas IL-6 and IL-10 showed no significant temporal changes. Notably, no consistent associations were observed between the dietary PUFA measures and the evaluated outcomes. Conclusions: Postoperative adaptation following sleeve gastrectomy differed across physiological domains, with progressive reductions in adiposity and CRP, an early decline followed by relative stabilization of RMR, and no detectable longitudinal changes in IL-6 or IL-10. Dietary PUFA measures showed no significant associations with postoperative outcomes.

## 1. Introduction

Since 1990s, obesity has increasingly been described as global epidemic, with prevalence rates continuing to rise worldwide [1,2]. Several meta-analyses have linked obesity to a higher risk of cardiovascular, cancer, liver dysfunction and musculoskeletal diseases [3,4,5,6]. Beyond excess body weight alone, obesity is currently understood as a chronic inflammatory state characterized by persistent yet low-grade systemic inflammation [7]. The biological relationship between inflammation and obesity remains only partly understood. On one side, obesity-related metabolic changes appear to induce a pro-inflammatory environment within adipose and muscle tissues [8,9,10]. At the same time, activated immune cells may contribute to insulin resistance, which can then drive fat accumulation [11,12,13]. These observations point to a multifactorial and likely bidirectional interaction between metabolic dysfunction and inflammation. Given this complexity, effective obesity management requires multifaceted therapeutic approaches.

Dietary modification and increased physical activity are typically the first-line approaches for weight reduction and obesity management [14]. Still, these strategies often result in only modest weight reduction and are frequently followed by weight regain over time [15]. This has been attributed to multifactorial physiological adaptations that favor energy conservation after weight loss, including reductions in resting metabolic rate, persistent hunger signaling, alterations in appetite-regulating hormones, and metabolic adaptations promoting increased energy efficiency [16,17,18]. In a large lifestyle intervention, only around half of the participants were able to maintain their weight loss after one year of follow-up [19]. Bariatric surgery has become one of the most effective treatment options for severe obesity, offering benefits that extend beyond weight reduction alone. In many patients, it leads to substantial improvement or remission of obesity-related metabolic disorders, particularly type 2 diabetes mellitus, alongside reductions in systemic inflammation and cardiometabolic risk [20,21]. Among bariatric procedures, sleeve gastrectomy is currently the most performed worldwide [22]. The procedure is associated with early and substantial changes in inflammatory markers, energy substrate utilization, fat distribution, and overall body composition [23]. Previous studies and meta-analyses have demonstrated significant postoperative reductions in inflammatory biomarkers such as C-reactive protein (CRP), interleukin-1β (IL-1β), and other pro-inflammatory cytokines, supporting important immunometabolic changes following metabolic surgery [24,25].

Although substantial reductions in body weight, adiposity, systemic inflammation, and resting energy expenditure following sleeve gastrectomy have been documented, the magnitude and timing of these physiological responses may differ across body-composition, metabolic, and inflammatory domains. Previous longitudinal studies have characterized changes in body composition, energy expenditure, and inflammatory or immune profiles over different postoperative intervals [26]. Resting energy expenditure also decreases substantially during postoperative weight loss, reflecting both the loss of metabolically active tissue and adaptive changes in energy metabolism [27]. Nevertheless, relatively few studies have concurrently characterized body composition, resting metabolic rate, and multiple inflammatory markers at closely spaced assessments during the period of rapid postoperative weight loss. Such repeated multidimensional assessment may provide a more integrated characterization of early postoperative adaptation and help determine whether changes in adiposity, energy metabolism, and systemic inflammation follow parallel or distinct temporal trajectories.

Beyond these longitudinal postoperative changes, dietary factors may contribute to interindividual variability in metabolic, inflammatory, and body-composition outcomes. Inflammation, lipid metabolism, and energy homeostasis are closely interconnected physiological processes, in which dietary fatty acid composition may play an important role [28,29]. Omega-6 and omega-3 polyunsaturated fatty acids (PUFAs) participate in pathways involved in lipid metabolism and inflammatory signaling [30]. Contemporary dietary patterns are often characterized by relatively high omega-6 and low omega-3 fatty acid intake. Because these fatty acids serve as precursors for lipid mediators with distinct and context-dependent roles in inflammatory regulation and resolution [31,32], their relative dietary balance has attracted interest in relation to obesity and metabolic health. However, the relationship between dietary omega-6/omega-3 balance and metabolic, inflammatory, and body-composition outcomes during the early postoperative period following sleeve gastrectomy remains insufficiently characterized.

Although postoperative changes in body composition, resting energy expenditure, and inflammation have been individually described, evidence from the Saudi population remains limited. Population-specific dietary patterns, including the amount and sources of omega-3 and omega-6 fatty acids, may influence dietary PUFA exposure and its potential relationships with postoperative outcomes. To our knowledge, no previous longitudinal study in Saudi adults has concurrently evaluated DXA-derived body composition, resting energy metabolism, multiple inflammatory biomarkers, and dietary PUFA intake following sleeve gastrectomy. Accordingly, the incremental contribution of the present pilot study lies in its integrated assessment of these domains within an understudied Saudi population. Therefore, the primary objective was to characterize the longitudinal trajectories of body composition, resting energy metabolism, and inflammatory biomarkers during the first nine months following sleeve gastrectomy. As a secondary exploratory objective, we examined whether the dietary omega-6/omega-3 ratio and individual omega-3 and omega-6 intakes were associated with concurrently measured body-composition, metabolic, and inflammatory outcomes. These outcomes included DXA-derived body-composition parameters, resting metabolic rate, respiratory quotient, CRP, IL-6, and IL-10, assessed at baseline and at 3, 6, and 9 months postoperatively.

## 2. Materials and Methods

### 2.1. Study Design and Setting

A prospective longitudinal pilot cohort study with a repeated-measures design was conducted. As this was an exploratory pilot longitudinal study, no formal a priori sample-size calculation was performed. The sample size was determined by the number of eligible participants who could be recruited during the study period. Of 63 individuals screened, 37 met the eligibility criteria and were enrolled. Participants were recruited from the Lifestyle and Health Research Center and the Tissue Biobank at Princess Nourah bint Abdulrahman University (PNU), Riyadh. Eligible individuals were identified through the pre-bariatric clinic at King Abdullah bin Abdulaziz University Hospital (KAAUH) following standardized presurgical evaluation protocols. Data were collected at four times: baseline (pre-surgery) and 3, 6, and 9 months after sleeve gastrectomy.

### 2.2. Participants

Inclusion criteria: willingness to complete all follow-up assessments; age 18–50 years; Saudi nationals residing in Riyadh; patients from the pre-bariatric clinic at KAAUH; BMI 35–40 kg/m^2^ with comorbidities or BMI > 40 kg/m^2^.

Exclusion criteria: previous bariatric surgery; pregnancy; acute or chronic conditions affecting energy expenditure; use of omega-3 fatty-acid or fish-oil supplements; use of weight-loss medications or medications known to influence inflammatory markers; chronic inflammatory diseases; and participation in another clinical trial.

Ethical considerations: ethical approval was granted by the Institutional Review Board (IRB) at KAAUH (24-0180). Written informed consent was obtained from all participants. Confidentiality and data protection were ensured according to the Declaration of Helsinki.

### 2.3. Anthropometric Measurements

Anthropometric measurements were obtained at all four study visits following standardized clinical protocols. A trained researcher measured height to the nearest 0.1 cm and weight to the nearest 50 g using calibrated Seca equipment (Seca GmbH & Co. KG, Hamburg, Germany), with participants standing barefoot and wearing light clothing. Body mass index (BMI) was calculated as weight in kilograms divided by height in meters squared. Waist circumference was assessed to the nearest 0.5 cm using a non-stretch measuring tape positioned at the midpoint between the lower rib margin and the iliac crest. The waist-to-height ratio was subsequently computed.

### 2.4. Body Composition Assessment

Dual-energy X-ray absorptiometry (DXA): Dual-energy X-ray absorptiometry (DXA; Lunar iDXA, GE Healthcare, Chicago, IL, USA) was used to obtain detailed whole-body measurements of body composition. Utilizing the enCORE software v18 platform, DXA provided estimates of bone mineral density, total and segmental body composition, and radiation exposure with minimal dose. The primary metrics derived included fat mass, fat-free mass, lean soft tissue mass, and bone mineral content. All scans were performed by a single trained technician following manufacturer guidelines. For individuals whose body size exceeded the table width, the validated mirroring technique was applied [33,34].

Resting metabolic rate: Resting metabolic rate (RMR) was assessed using indirect calorimetry with a ventilated umbrella hood (Metamax analyzer, Cortex, Leipzig, Germany). Participants lay supine for a minimum of 20 min to ensure steady-state readings. Standardization required 7–8 h of sleep, a 12 h fast, avoidance of vigorous physical activity for at least 48 h, and abstinence from caffeine for 24 h prior to each assessment [35].

### 2.5. Blood Sample Collection and Biochemical Analysis

Fasting blood samples were collected at all visits for measurement of CRP, IL-6, and IL-10. These were selected to provide complementary assessment of systemic inflammatory status. CRP represents a widely used marker of systemic low-grade inflammation, IL-6 reflects a key pro-inflammatory pathway relevant to obesity-associated metabolic inflammation, and IL-10 represents an anti-inflammatory regulatory pathway. Plasma samples were obtained from EDTA tubes after centrifugation at 3000 rpm for 15 min at 4 °C, aliquoted, and stored at −80 °C. IL-6 and IL-10 were quantified in duplicate using human ELISA kits (Solarbio Life Sciences, Beijing, China), with four-parameter logistic standard curves and optical density measurement at 450 nm.

### 2.6. Dietary Intake Assessment

Dietary intake was assessed using three-day food records [two weekdays, one weekend day]. Participants documented all foods and beverages consumed, including portion sizes, preparation methods, and brand names. Records were reviewed with participants and entered into ESHA’s Food Processor^®^ Nutrition Analysis Software [version 11.15.94], which provided standardized nutrient calculations, including omega-6 and omega-3 fatty acid quantification. Glycemic index and glycemic load were calculated using established reference tables [36,37,38]. No study-specific dietary counseling was provided to participants during follow-up; therefore, the dietary records reflected participants’ self-selected postoperative dietary intake.

### 2.7. Statistics

Descriptive statistics were presented as mean ± standard deviation (SD) for continuous variables and as frequencies with percentages for categorical variables. All statistical analyses were performed using IBM SPSS Statistics (Version 21.0; IBM Corp., Armonk, NY, USA). Statistical significance was set at alpha = 0.05 for all analyses.

To examine longitudinal changes in body composition, resting metabolic rate (RMR), respiratory quotient (RQ), and inflammatory biomarkers across study visits, and to assess the independent associations of dietary omega-6/omega-3 fatty acid ratio with these outcomes linear mixed models (LMM) were performed. In these models, the outcome variable was specified as the dependent variable. Visit number (timepoint: 1–4) was entered as a categorical fixed factor representing the preoperative baseline assessment (Visit 1) and three postoperative follow-up visits (Visits 2–4). The dietary predictor was entered as a continuous covariate. Age (continuous), sex (male/female), and presence of comorbidity (yes/no) were included as additional fixed covariates to adjust for potential confounding. Age and sex were included because of their established relationships with body composition, resting energy expenditure, and inflammatory profiles, while comorbidity status was included to account for underlying metabolic and clinical heterogeneity within the cohort.

A first-order autoregressive (AR(1)) covariance structure was specified for the repeated measures, assuming that correlations between repeated observations decrease as the interval between assessments increases. Missing follow-up observations due to loss to follow-up were not imputed. The LMMs incorporated all available repeated observations for each participant, allowing participants with incomplete follow-up to contribute data from the visits they attended. A sensitivity analysis was conducted to evaluate the necessity of random effects. For each outcome, two models were compared: a fixed-effects-only model and a model additionally incorporating a random intercept for patient ID, both using AR (1) covariance. Model fit was evaluated using the −2 Log Likelihood (−2LL) statistic and Akaike Information Criterion (AIC). The difference in −2LL between models follows a Chi-square distribution with degrees of freedom equal to the difference in estimated parameters. For the primary outcome (Total Body Fat %), the −2LL was identical across both models (649.953), yielding a Chi-square of 0.000 (df = 1, *p* = 1.000), indicating that the random intercept contributed no additional explanatory variance. Therefore, random effects were excluded from all final models in accordance with the principle of parsimony.

## 3. Results

### 3.1. Baseline Characteristics

**Figure 1:**
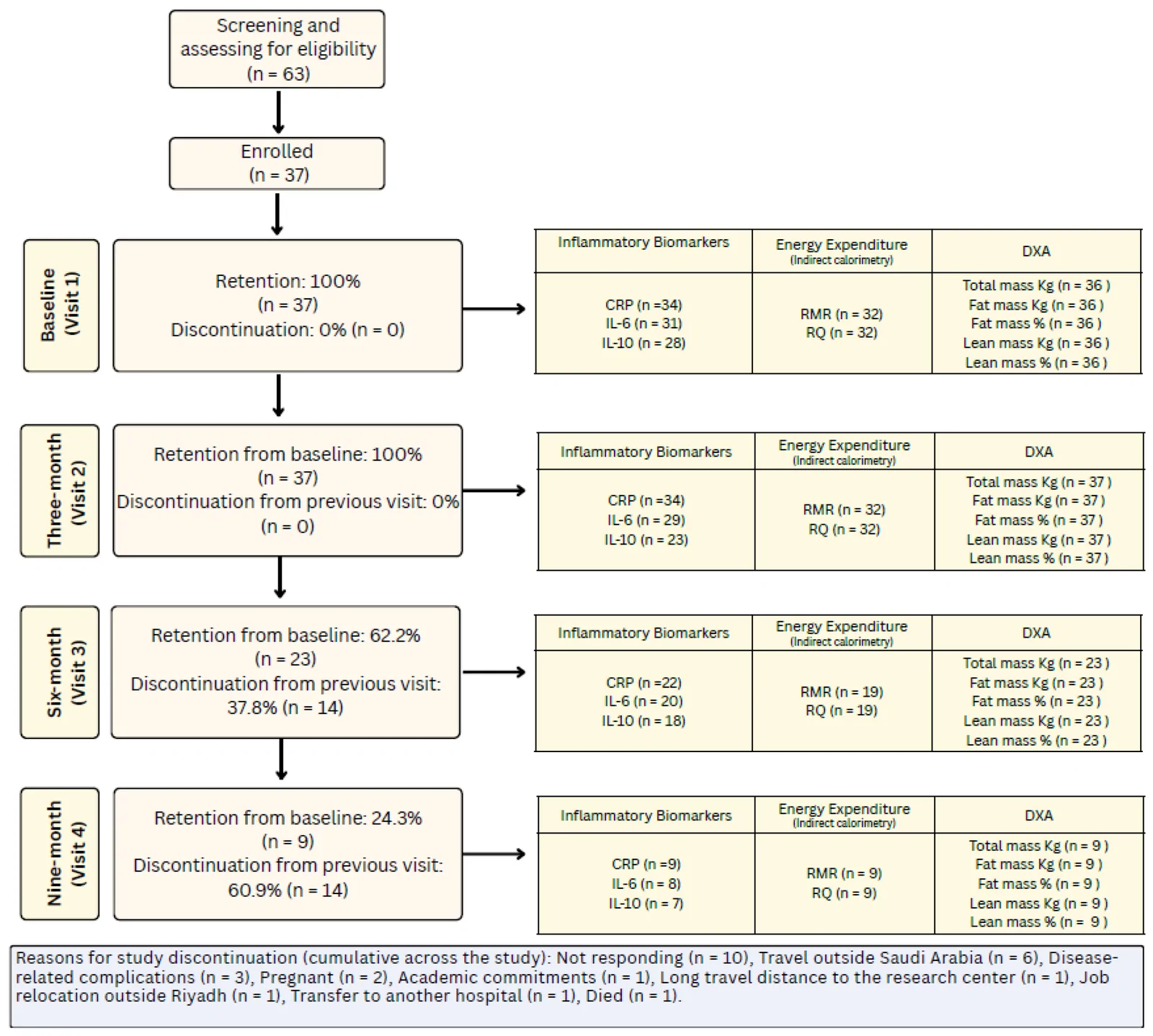
Participant flow and retention across the 9-month follow-up period. Percentages of participants assessed at each visit are calculated relative to the baseline cohort (n = 37), whereas loss to follow-up is calculated relative to the preceding visit. Overall retention at 9 months was 24.3% (9/37).

As shown in Table 1, the study included 37 participants with a mean age of 35.11 ± 11.30 years and a mean BMI of 43.76 ± 6.44 kg/m^2^. Females represented 67.6% of the cohort, while males accounted for 32.4%. Comorbidities were present in 40.5% of participants. All participants completed the baseline and second visits, whereas 23 participants attended the third visit and 9 completed the fourth visit (Figure 1). To assess potential attrition bias, baseline characteristics were compared between participants who completed the 9-month follow-up (n = 9) and those who did not (n = 28) (Supplementary Table S1). No statistically significant baseline differences were observed between completers and non-completers in age, BMI, body-composition measures, RMR, inflammatory biomarkers, or dietary omega-3, omega-6, and omega-6/omega-3 ratio measures (all *p* > 0.05).

### 3.2. Longitudinal Changes in Body Composition, Resting Metabolic Rate, and Inflammatory Biomarkers: Omega-6/Omega-3 Ratio Model

**Figure 2:**
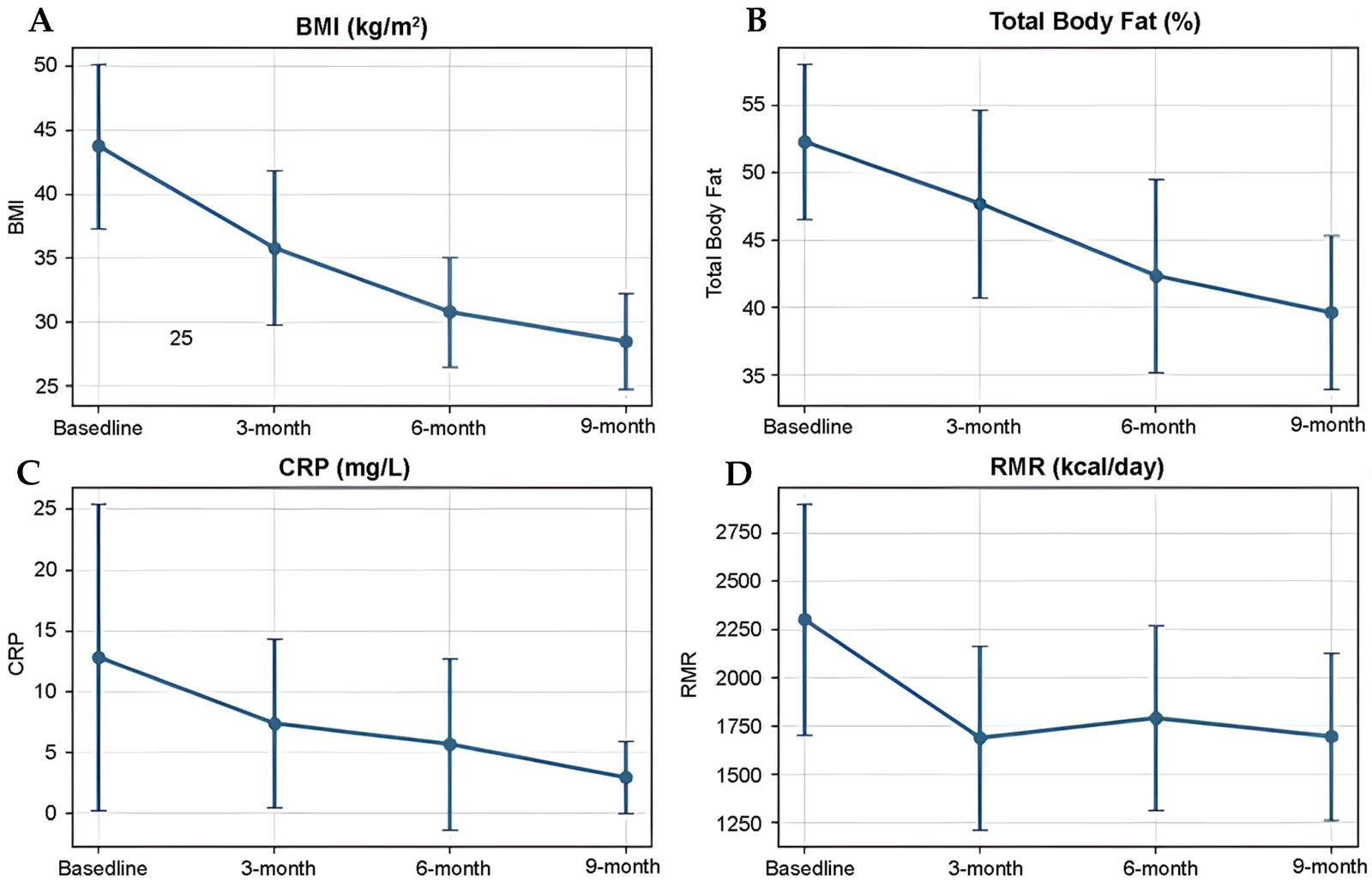
Longitudinal changes in (**A**) BMI, (**B**) total body fat percentage, (**C**) C-reactive protein (CRP), and (**D**) resting metabolic rate (RMR) from baseline to 9 months following sleeve gastrectomy. Data are presented as mean ± SD. Outcome-specific sample sizes at each visit are reported in Figure 1. Statistical comparisons across visits were performed using linear mixed models.

Dietary omega-3 intake, omega-6 intake, and the omega-6/omega-3 ratio did not change significantly across study visits. Mean omega-3 intake was 0.468 ± 0.381 g/day at baseline and 0.341 ± 0.263 g/day at 9 months (*p* = 0.271 for time), while mean omega-6 intake was 3.942 ± 3.728 g/day and 2.897 ± 2.302 g/day, respectively (*p* = 0.485). The corresponding omega-6/omega-3 ratios were 10.61 ± 7.19 and 10.26 ± 7.44 (*p* = 0.506). Values for all study visits are presented in Supplementary Table S2. In contrast, significant longitudinal changes were observed across study visits for most body composition and metabolic variables (Table 2). BMI decreased progressively from 43.76 ± 6.44 kg/m^2^ at baseline to 28.49 ± 3.77 kg/m^2^ at Visit 4 (Figure 2A, *p* < 0.001 for time). Similarly, total body fat percentage declined from 52.32 ± 5.76% to 39.62 ± 5.71% (Figure 2B, *p* < 0.001). Similarly, fat mass, total body mass, and body weight declined significantly over time (all *p* < 0.001). Absolute lean mass also decreased significantly across visits (*p* < 0.001), whereas lean body mass percentage increased from 46.55 ± 5.48% at baseline to 58.30 ± 5.31% at Visit 4 (*p* < 0.001).

Resting metabolic rate (RMR) changed significantly across visits (Figure 2D, *p* < 0.001), with a marked reduction from 2303.16 ± 597.98 kcal/day at baseline to 1689.35 ± 476.47 kcal/day at Visit 2, followed by relatively smaller changes at subsequent visits. Among the inflammatory biomarkers, CRP declined from 12.84 ± 12.60 mg/L at baseline to 2.96 ± 2.95 mg/L at Visit 4, with a significant overall effect of time (Figure 2C, *p* = 0.001). In contrast, IL-6 and IL-10 did not demonstrate significant longitudinal changes (*p* = 0.922 and *p* = 0.683, respectively).

After adjustment for age, gender, time, and comorbidity, the omega-6/omega-3 ratio was not significantly associated with any of the examined body composition, metabolic, or inflammatory outcomes. Specifically, the associations with total body fat percentage (β = −0.033, 95% CI: −0.172 to 0.106, *p* = 0.640) and lean body mass percentage (β = 0.012, 95% CI: −0.129 to 0.152, *p* = 0.869) were not significant. Similarly, no significant independent associations were observed between the omega-6/omega-3 ratio and fat mass, absolute lean mass, total body mass, body weight, BMI, RMR, RQ, IL-6, IL-10, or CRP (all *p* > 0.05).

### 3.3. Comparison of Dietary Fatty Acid Predictors

Table 3 presents the comparative associations of the omega-6/omega-3 ratio, omega-3 intake, and omega-6 intake with body composition, metabolic, and inflammatory outcomes. Neither the omega-6/omega-3 ratio nor omega-6 intake was significantly associated with any of the examined outcomes (all *p* > 0.05). Similarly, omega-3 intake was not significantly associated with body composition measures, RMR, RQ, IL-6, or IL-10. However, omega-3 intake was positively associated with CRP levels (β = 4.506, 95% CI: 0.223 to 8.789, *p* = 0.039). No corresponding association with CRP was observed for the omega-6/omega-3 ratio (β = 0.128, 95% CI: −0.310 to 0.566, *p* = 0.563) or omega-6 intake (β = 0.062, 95% CI: −0.118 to 0.242, *p* = 0.495).

Standardized effect estimates were generally small across dietary predictor–outcome associations (Supplementary Table S3), with absolute standardized β values ranging from 0.001 to 0.201. The largest standardized effect was observed for the association between omega-3 intake and CRP (standardized β = 0.201), whereas all remaining standardized coefficients were <0.10.

### 3.4. Post Hoc Pairwise Comparisons Between Visits: Omega-6/Omega-3 Ratio Model

Post hoc pairwise comparisons demonstrated continued postoperative changes in several body composition measures (Table 4). Total body fat percentage differed significantly between Visits 2 and 3 (β = 5.573, 95% CI: 3.852 to 7.294, *p* < 0.001), Visits 2 and 4 (β = 8.885, 95% CI: 5.788 to 11.983, *p* < 0.001), and Visits 3 and 4 (β = 3.312, 95% CI: 0.610 to 6.014, *p* = 0.008). Similarly, fat mass showed significant differences across all postoperative pairwise comparisons, including between Visits 3 and 4 (β = 5.727, 95% CI: 0.455 to 11.000, *p* = 0.026). Lean body mass percentage also differed significantly across all postoperative visits (all *p* ≤ 0.009).

Total body mass, body weight, and BMI differed significantly between Visits 2 and 3 and between Visits 2 and 4 (all *p* < 0.001), whereas the corresponding differences between Visits 3 and 4 were not statistically significant, suggesting relative stabilization of these measures during the later postoperative period. Absolute lean mass did not differ significantly between postoperative visits.

No significant postoperative pairwise differences were observed for RMR, RQ, IL-6, IL-10, or CRP after Bonferroni correction (all *p* > 0.05).

## 4. Discussion

The present longitudinal pilot study characterized changes in body composition, resting metabolic rate, and inflammatory biomarkers during the first nine months following sleeve gastrectomy, while secondarily exploring their associations with dietary PUFA balance. The principal findings demonstrated distinct postoperative trajectories across physiological domains. Body weight, BMI, and adiposity declined progressively throughout follow-up, whereas resting metabolic rate decreased predominantly during the early postoperative period and subsequently remained relatively stable. The inflammatory response was also marker-specific, with a progressive reduction in CRP, while IL-6 and IL-10 showed no significant longitudinal changes. Collectively, these findings indicate that postoperative changes in body composition, energy metabolism, and systemic inflammation do not necessarily occur in parallel. In secondary exploratory analyses, the dietary omega-6/omega-3 ratio was not significantly associated with body-composition, metabolic, or inflammatory outcomes. Supplementary analyses of individual fatty acids similarly showed limited associations, although omega-3 intake was positively associated with CRP. Given the small effect estimates, exploratory nature of these analyses, and substantial attrition, the dietary findings should be interpreted cautiously and require confirmation in larger, adequately powered longitudinal studies.

Consistent with previous bariatric surgery literature, marked reductions in body weight, BMI, and adiposity were observed throughout the postoperative follow-up period. Although substantial changes were evident during the earlier postoperative assessments, body-composition remodeling continued during later follow-up, with significant reductions in total body fat percentage and fat mass persisting between the later visits. Absolute lean mass also declined over time, whereas lean body mass percentage increased, indicating that the relative contribution of lean tissue to total body mass increased as adiposity decreased. Previous longitudinal studies have similarly demonstrated substantial reductions in body weight and fat mass during the first postoperative year, with the rate of weight loss generally becoming less pronounced as follow-up progresses [26]. Importantly, the present study incorporated repeated DXA assessments alongside metabolic and inflammatory measurements, allowing changes in body composition to be considered within the broader pattern of postoperative physiological adaptation. These findings highlight the dynamic nature of body-composition remodeling during the first nine months following sleeve gastrectomy and provide an important physiological context for interpreting the accompanying changes in resting energy metabolism and inflammatory status.

A distinct temporal pattern was also observed for resting metabolic rate. RMR declined markedly during the early postoperative period, with the largest reduction occurring between baseline and the first postoperative assessment, followed by relative stabilization at subsequent visits. This pattern is consistent with previous studies demonstrating substantial reductions in resting energy expenditure during the initial months following bariatric surgery, when weight loss and changes in body composition are most pronounced [27]. Such reductions are likely to reflect, at least in part, the loss of metabolically active tissue accompanying rapid weight loss, although adaptive reductions in energy expenditure beyond those expected from changes in body size and composition have also been described [39]. Interestingly, the absence of further significant reductions between the later postoperative visits in the present study suggests that the major adjustment in resting energy expenditure occurred relatively early, despite continued changes in body weight and composition. This apparent divergence between the trajectories of RMR and body composition supports the concept that postoperative physiological adaptations may occur at different rates across metabolic domains. Long-term studies have similarly suggested that metabolic adaptation may become less pronounced as weight loss stabilizes following sleeve gastrectomy [40]. Collectively, these findings highlight the early postoperative period as a particularly dynamic phase of energy-metabolic adaptation following sleeve gastrectomy.

The inflammatory response demonstrated a different and marker-specific temporal pattern. CRP decreased significantly across the postoperative period, with reductions already evident at the first follow-up assessment and persisting through nine months. This finding is consistent with previous studies and meta-analyses demonstrating substantial reductions in CRP following bariatric surgery, likely reflecting the attenuation of the chronic low-grade systemic inflammatory state associated with obesity [24,25]. In contrast, IL-6 and IL-10 did not show significant longitudinal changes across the four assessments. Previous studies have reported variable responses of individual cytokines following bariatric surgery. For example, significant reductions in both CRP and IL-6 have been observed as early as three months following sleeve gastrectomy [41], whereas other longitudinal studies evaluating broader inflammatory profiles have demonstrated that individual inflammatory mediators differ in both the magnitude and timing of their postoperative responses [42]. Considerable between-study heterogeneity has also been reported in meta-analytic evaluations of postoperative inflammatory biomarkers [24]. Together, these findings suggest that improvement in systemic inflammation following sleeve gastrectomy may not be reflected uniformly across individual inflammatory mediators. The marked decline in CRP alongside the absence of significant longitudinal changes in IL-6 and IL-10 in the present study therefore supports a marker-specific inflammatory response during the early postoperative period.

Beyond the longitudinal postoperative changes, secondary exploratory analyses provided limited evidence for independent associations between dietary PUFA measures and the evaluated outcomes. After adjustment for age, sex, comorbidity, and time, the dietary omega-6/omega-3 ratio was not significantly associated with body composition, body weight, BMI, resting metabolic rate, or the measured inflammatory biomarkers. Similarly, omega-6 intake showed no significant associations with any evaluated outcome, while omega-3 intake was unrelated to body-composition and metabolic measures. An isolated positive association was observed between omega-3 intake and CRP. This finding contrasts with much of the existing literature, in which omega-3 PUFA supplementation has generally been associated with reductions in CRP, although inflammatory responses to omega-3 appear heterogeneous across populations and clinical contexts [43]. Given its modest standardized effect, absence of corresponding associations with IL-6 or IL-10, and the exploratory nature of the analysis, this isolated finding should be interpreted cautiously and should not be considered evidence of a pro-inflammatory effect of omega-3 intake.

The lack of significant associations with body composition contrasts with some observational evidence linking PUFA intake patterns to adiposity-related phenotypes. Torres-Castillo et al. reported that a higher dietary omega-6/omega-3 ratio was associated with greater body fat percentage and waist circumference, as well as higher insulin and HOMA-IR [44]. More recently, population-based evidence has also identified associations between omega-6 PUFA profiles and measures of adiposity and metabolic health [45]. Several biological pathways may provide a plausible context for these observations. PUFAs participate in the regulation of adipocyte differentiation, lipid storage, and lipid metabolism through transcriptional pathways involving peroxisome proliferator-activated receptors (PPARs), sterol regulatory element-binding proteins (SREBPs), and liver X receptors (LXRs) [46]. However, such mechanisms do not establish an independent effect of dietary PUFA exposure on postoperative body-composition remodeling. The absence of comparable associations in the present cohort may reflect the distinct physiological context of rapid surgically associated weight loss, as well as differences in population characteristics, dietary assessment, sample size, and follow-up duration.

With respect to inflammation, the limited dietary associations are noteworthy given the established involvement of PUFAs in inflammatory signaling [47,48,49]. The pronounced postoperative decline in CRP occurred without detectable associations with the omega-6/omega-3 ratio or omega-6 intake, while IL-6 and IL-10 showed no significant associations with any dietary PUFA measure. This pattern suggests that the postoperative inflammatory trajectory was more prominent than measurable relationships with dietary PUFA exposure in this cohort. The absence of associations with IL-6 and IL-10 may also reflect their substantial interindividual variability and the reduced sample size at later follow-up assessments. Moreover, circulating CRP, IL-6, and IL-10 may not fully capture tissue-specific inflammatory responses, while dietary fatty-acid intake estimated from three-day food records may not accurately reflect biologically relevant PUFA exposure.

Several limitations should be considered when interpreting these findings. This pilot study had a small baseline sample (n = 37) and substantial attrition, with only nine participants completing the 9-month follow-up, limiting statistical power and increasing the potential for attrition bias despite no significant baseline differences between completers and non-completers. Missing follow-up observations were not imputed, although LMMs incorporated all available repeated measurements. Residual confounding remains possible because physical activity, use of vitamin, mineral, or other dietary supplements, and total energy intake were not included in the adjusted models. Dietary assessment using three-day food records is susceptible to reporting error and under-reporting, while fatty-acid estimates depend on nutrient-database accuracy. Longitudinal total energy and macronutrient intake data were unavailable, limiting assessment of broader postoperative dietary changes. Finally, multiple exploratory analyses, substantial biomarker variability, and the limited inflammatory panel warrant cautious interpretation, particularly of the isolated omega-3–CRP association. These findings should therefore be considered exploratory and require confirmation in larger longitudinal studies with more complete follow-up.

## 5. Conclusions

Overall, sleeve gastrectomy was associated with substantial changes that followed distinct temporal patterns across body composition, body weight, resting metabolic rate, and systemic inflammation. Specifically, body weight, adiposity, and CRP decreased progressively during the first nine postoperative months, whereas RMR declined markedly during the early postoperative period and subsequently showed relative stabilization. The dietary omega-6/omega-3 ratio and individual omega-3 and omega-6 intakes showed no consistent associations with the evaluated outcomes. Future larger longitudinal studies incorporating objective fatty acid biomarkers and controlled dietary interventions are warranted to further clarify the role of dietary PUFA balance in postoperative metabolic adaptation following bariatric surgery.

## Figures and Tables

**Table 1:** Baseline demographic and clinical characteristics (n = 37).

Characteristics		n (%)
Age:	Mean ± SD	35.11 ± 11.30
Gender:	Female	25 (67.6%)
	Male	12 (32.4%)
BMI:	Mean ± SD	43.76 ± 6.44
Comorbidity:	Yes	15 (40.5%)
	No	22 (59.5%)
Number of visits:	Baseline	37 (100%)
	Second visit	37 (100%)
	Third visit	23 (62.2%)
	Fourth visit	9 (24.3%)

Data are presented as mean ± standard deviation (SD) or number (%). Percentages for follow-up visits were calculated based on the total study cohort (n = 37).

**Table 2:** Body composition, RMR, and inflammatory biomarkers omega-3/6 ratio model.

Factor	Visit 1 Mean ± SD	Visit 2 Mean ± SD	Visit 3 Mean ± SD	Visit 4 Mean ± SD	Ratio B (SE)	95% CI (Ratio)	Ratio	Age	Gender	Time	Comorbidity
Total Body Fat (%)	52.32 ± 5.76	47.72 ± 6.97	42.38 ± 7.18	39.62 ± 5.71	−0.033 (0.070)	−0.172, 0.106	0.640	0.054	0.001 *	<0.001 *	0.395
Fat Mass (kg)	58.34 ± 16.68	44.38 ± 13.53	35.10 ± 10.04	28.59 ± 6.08	−0.235 (0.134)	−0.503, 0.033	0.084	0.312	0.643	<0.001 *	0.634
Lean Body Mass (%)	46.55 ± 5.48	50.53 ± 6.60	55.59 ± 6.71	58.30 ± 5.31	0.012 (0.070)	−0.129, 0.152	0.869	0.057	0.001 *	<0.001 *	0.406
Lean Mass (kg)	52.52 ± 10.33	47.35 ± 9.41	47.00 ± 10.18	44.37 ± 12.49	−0.134 (0.087)	−0.309, 0.040	0.130	0.214	<0.001 *	<0.001 *	0.541
Total Body Mass (kg)	113.84 ± 23.77	94.72 ± 20.52	84.75 ± 17.08	75.53 ± 17.35	−0.284 (0.191)	−0.666, 0.098	0.143	0.794	0.007 *	<0.001 *	0.893
Body Weight (kg)	116.95 ± 23.08	95.50 ± 20.83	83.25 ± 16.90	75.71 ± 17.37	0.019 (0.153)	−0.285, 0.324	0.899	0.812	0.003 *	<0.001 *	0.505
BMI (kg/m^2^)	43.76 ± 6.44	35.81 ± 6.05	30.80 ± 4.27	28.49 ± 3.77	0.027 (0.058)	−0.089, 0.143	0.642	0.335	0.608	<0.001 *	0.174
RMR (kcal/day)	2303.16 ± 597.98	1689.35 ± 476.47	1792.11 ± 478.14	1696.33 ± 432.54	−13.013 (12.623)	−38.142, 12.117	0.306	0.828	<0.001 *	<0.001 *	0.922
RQ	0.75 ± 0.047	0.72 ± 0.068	0.71 ± 0.047	0.73 ± 0.053	−0.0004 (0.0014)	−0.0032, 0.0025	0.796	0.748	0.375	0.011 *	0.949
IL-6 (pg/mL)	29.54 ± 40.63	28.35 ± 56.91	35.02 ± 66.63	47.45 ± 81.00	0.597 (0.838)	−1.078, 2.273	0.479	0.064	0.202	0.922	0.037 *
IL-10 (pg/mL)	36.72 ± 24.58	39.79 ± 28.24	37.18 ± 32.75	45.24 ± 21.45	−0.225 (0.450)	−1.130, 0.679	0.619	0.086	0.229	0.683	0.631
CRP (mg/L)	12.84 ± 12.60	7.41 ± 6.93	5.70 ± 7.04	2.96 ± 2.95	0.128 (0.220)	−0.310, 0.566	0.563	0.063	0.576	0.001 *	0.011 *

* *p* < 0.05. Bonferroni correction was applied for multiple comparisons. Adjusted for age, sex, time and comorbidity. LMM = linear mixed model, unstructured covariance. n = V1/V2/V3/V4.

**Table 3:** Comparison of dietary fatty acid predictors: omega-3/6 ratio vs. omega-3 (g) vs. omega-6 (g).

Factor	Omega Ratio β (95% CI)	*p*-Value	Omega-3 (g) β (95% CI)	*p*-Value	Omega-6 (g) β (95% CI)	*p*-Value
Total Body Fat (%)	−0.033 (−0.172, 0.106)	0.640	−0.284 (−1.673, 1.105)	0.684	−0.012 (−0.065, 0.042)	0.671
Fat Mass (kg)	−0.235 (−0.503, 0.033)	0.084	0.139 (−2.615, 2.892)	0.920	−0.011 (−0.117, 0.095)	0.837
Lean Body Mass (%)	0.012 (−0.129, 0.152)	0.869	−0.251 (−1.652, 1.151)	0.722	−0.001 (−0.055, 0.054)	0.975
Lean Mass (kg)	−0.134 (−0.309, 0.040)	0.130	0.524 (−1.247, 2.295)	0.557	0.002 (−0.067, 0.071)	0.959
Total Body Mass (kg)	−0.284 (−0.666, 0.098)	0.143	1.342 (−2.544, 5.227)	0.493	0.005 (−0.145, 0.155)	0.945
Body Weight (kg)	0.019 (−0.285, 0.324)	0.899	0.960 (−2.098, 4.018)	0.533	0.016 (−0.101, 0.133)	0.784
BMI (kg/m^2^)	0.027 (−0.089, 0.143)	0.642	0.313 (−0.846, 1.472)	0.592	0.007 (−0.037, 0.052)	0.743
RMR (kcal/day)	−13.013 (−38.142, 12.117)	0.306	101.437 (−128.040, 330.913)	0.382	1.105 (−9.355, 11.566)	0.834
RQ	−0.0004 (−0.0032, 0.0025)	0.796	0.0027 (−0.0235, 0.0289)	0.837	0.0003 (−0.0008, 0.0014)	0.614
IL-6 (pg/mL)	0.597 (−1.078, 2.273)	0.479	−2.430 (−18.995, 14.135)	0.770	−0.025 (−0.659, 0.610)	0.938
IL-10 (pg/mL)	−0.225 (−1.130, 0.679)	0.619	−2.486 (−11.481, 6.508)	0.581	−0.146 (−0.482, 0.190)	0.385
CRP (mg/L)	0.128 (−0.310, 0.566)	0.563	4.506 (0.223, 8.789)	0.039 *	0.062 (−0.118, 0.242)	0.495

β = unstandardized regression coefficient. * *p* < 0.05. Bonferroni correction applied for post hoc comparisons (not applicable to these omnibus predictor tests). First-order autoregressive AR(1) covariance (per supervisor guidance). Adjusted for age, sex, comorbidity, and time.

**Table 4:** Post hoc pairwise comparisons between visits—omega-3/6 ratio model (Bonferroni correction).

Factor	V2 vs. V3 β (95% CI)	*p*-Value	V2 vs. V4 β (95% CI)	*p*-Value	V3 vs. V4 β (95% CI)	*p*-Value
Total Body Fat (%)	5.573 (3.852, 7.294)	<0.001 *	8.885 (5.788, 11.983)	<0.001 *	3.312 (0.610, 6.014)	0.008 *
Fat Mass (kg)	9.196 (5.855, 12.538)	<0.001 *	14.924 (8.819, 21.028)	<0.001 *	5.727 (0.455, 11.000)	0.026 *
Lean Body Mass (%)	−5.399 (−7.134, −3.665)	<0.001 *	−8.721 (−11.825, −5.618)	<0.001 *	−3.322 (−6.040, −0.604)	0.009 *
Lean Mass (kg)	0.142 (−2.015, 2.299)	1.000	0.862 (−2.998, 4.722)	1.000	0.720 (−2.661, 4.100)	1.000
Total Body Mass (kg)	9.359 (4.601, 14.117)	<0.001 *	15.696 (7.034, 24.359)	<0.001 *	6.338 (−1.161, 13.836)	0.148
Body Weight (kg)	11.686 (7.865, 15.508)	<0.001 *	16.913 (9.890, 23.935)	<0.001 *	5.226 (−0.815, 11.268)	0.130
BMI (kg/m^2^)	4.415 (2.972, 5.858)	<0.001 *	6.150 (3.520, 8.779)	<0.001 *	1.735 (−0.540, 4.010)	0.253
RMR (kcal/day)	−93.452 (−429.040, 242.137)	1.000	−47.505 (−503.795, 408.786)	1.000	45.947 (−412.488, 504.381)	1.000
RQ	0.014 (−0.024, 0.051)	1.000	−0.013 (−0.065, 0.040)	1.000	−0.027 (−0.077, 0.024)	0.941
IL-6 (pg/mL)	−3.528 (−25.183, 18.128)	1.000	−3.760 (−39.007, 31.487)	1.000	−0.232 (−31.171, 31.636)	1.000
IL-10 (pg/mL)	0.073 (−12.337, 12.482)	1.000	8.242 (−12.262, 28.745)	1.000	8.169 (−10.413, 26.751)	1.000
CRP (mg/L)	2.178 (−3.268, 7.623)	1.000	5.471 (−2.692, 13.634)	0.444	3.293 (−4.644, 11.230)	1.000

β represents the estimated unstandardized mean difference between visits. * *p* < 0.05. *p*-values were adjusted for multiple pairwise comparisons using the Bonferroni method. V1 = baseline (preoperative); V2 = 3-month follow-up; V3 = 6-month follow-up; V4 = 9-month follow-up. Pairwise comparisons were derived from the linear mixed model including the omega-6/omega-3 ratio, time, age, sex, and comorbidity. An unstructured covariance matrix was used.

## Data Availability

The data presented in this study are available from the corresponding author upon reasonable request. The data are not publicly available due to privacy and ethical restrictions.

## Supplementary Materials

The following supporting information can be downloaded at: https://www.mdpi.com/article/10.3390/metabo16090664/s1, Supplementary Table S1. Baseline characteristics of participants who completed versus did not complete the 9-month follow-up. Data are presented as median (interquartile range [IQR]). Completers were defined as participants who attended the 9-month follow-up (Visit 4), whereas non-completers were those who did not attend the 9-month assessment. Between-group comparisons were performed using the Mann–Whitney U test unless otherwise indicated. Sample sizes are reported for each variable where applicable. BMI, body mass index; RMR, resting metabolic rate; CRP, C-reactive protein; IL, interleukin; Supplementary Table S2. Dietary Omega-3 and Omega-6 Intake and Omega-6/Omega-3 Ratio Across Study Visits: Data are presented as mean ± SD. Omega-3 and omega-6 intakes are expressed as g/day. Time *p*-values were obtained using linear mixed models (LMMs). V1 = baseline (preoperative); V2 = 3-month follow-up; V3 = 6-month follow-up; V4 = 9-month follow-up. SD = standard deviation; A *p*-value < 0.05 was considered statistically significant; Supplementary Table S3: Standardized effect sizes for dietary predictor–outcome associations. Standardized coefficients (standardized β), representing the SD-unit change in the outcome per SD-unit change in the predictor, were calculated for all 36 predictor–outcome combinations using pooled sample standard deviations across all observed timepoints. Across all combinations, standardized effects ranged from 0.001 to 0.201 in absolute value. The single statistically significant association identified in this analysis, omega-3 intake with CRP, corresponds to a standardized B of 0.201, a small-to-modest effect by conventional benchmarks [50]. All remaining associations had standardized effects below 0.1, indicating negligible clinical magnitude consistent with their non-significant *p*-values.
