# Supplementary material for: Longitudinal Changes in Body Composition, Resting Metabolic Rate, and Inflammation After Sleeve Gastrectomy: Associations with Dietary Polyunsaturated Fatty Acid Balance
Source: Metabolites. 2026 Sep 9;16(9):664. doi: 10.3390/metabo16090664 (PMC13609386; doi:10.3390/metabo16090664)
Supplement: Supplementary file 1 [file metabolites-16-00664-s001.zip › metabolites-4494861-supplementary.pdf]

Supplementary Table S1. Baseline characteristics of participants who completed versus did not complete the 9-month follow-up. Data are presented as median (interquartile range [IQR]). Completers were defined as participants who attended the 9-month follow-up (Visit 4), whereas non-completers were those who did not attend the 9-month assessment. Between-group comparisons were performed using the Mann–Whitney U test unless otherwise indicated. Sample sizes are reported for each variable where applicable. BMI, body mass index; RMR, resting metabolic rate; CRP, C-reactive protein; IL, interleukin.

**Baseline characteristic**	**Completers**	**Non-completers**	***P* value**
Age, years	32.00 (24.00–40.50)	27.50 (23.00–40.50)	0.571
BMI, kg/m^2	40.04 (37.85–47.84)	44.10 (40.90–48.03)	0.136
Fat mass, kg	48.74 (47.67–66.79)	59.90 (51.36–79.19)	0.148
Total body mass, kg	98.70 (91.70–124.60)	113.50 (99.30–140.20)	0.282
RMR, kcal/day	2215 (1886–2762)	2195 (2017–2567)	0.991
CRP, mg/L	19.00 (12.40–30.10)	13.20 (6.35–22.85)	0.390
IL-6, pg/mL	27.86 (21.29–68.27)	19.22 (12.86–26.27)	0.078
IL-10, pg/mL	29.59 (25.82–82.13)	31.24 (22.96–38.07)	0.793
Omega-3 intake, g/day	0.547 (0.313–1.210)	0.442 (0.174–0.779)	0.341
Omega-6 intake, g/day	5.13 (2.06–12.70)	3.29 (1.34–4.80)	0.306
Omega-6/omega-3 ratio	8.29 (6.37–9.74)	7.08 (5.97–9.17)	0.340

Abbrevation: BMI, body mass index; RMR, resting metabolic rate; CRP, C-reactive protein; IL, interleukin. Footnotes: Data are presented as median (interquartile range [IQR]). Completers were defined as participants who attended the 9-month follow-up (Visit 4), whereas non-completers were participants who did not attend the 9-month assessment. Between-group comparisons were performed using the Mann–Whitney U test.

Supplementary Table S2. Dietary Omega-3 and Omega-6 Intake and Omega-6/Omega-3 Ratio Across Study Visits: Data are presented as mean ± SD. Omega-3 and omega-6 intakes are expressed as g/day. Time *p*-values were obtained using linear mixed models (LMMs). V1 = baseline (preoperative); V2 = 3-month follow-up; V3 = 6-month follow-up; V4 = 9-month follow-up. SD = standard deviation; A *p*-value < 0.05 was considered statistically significant.

**Factor**	Visit 1	Visit 2	Visit 3	Visit 4	*P* value
Omega-3(g)	0.468±0.381	0.320±0.408	0.433±0.567	0.341±0.263	0.271
Omega-6(g)	3.942±3.728	2.711±3.811	6.331±17.274	2.897±2.302	0.485
Omega Ratio	7.962±2.951	8.787±2.943	9.399±5.811	8.471±2.573	0.506

Supplementary Table S3: Standardized effect sizes for dietary predictor–outcome associations. Standardized coefficients (standardized β), representing the SD-unit change in the outcome per SD-unit change in the predictor, were calculated for all 36 predictor–outcome combinations using pooled sample standard deviations across all observed timepoints. Across all combinations, standardized effects ranged from 0.001 to 0.201 in absolute value. The single statistically significant association identified in this analysis, omega-3 intake with CRP, corresponds to a standardized B of 0.201, a small-to-modest effect by conventional benchmarks [50]. All remaining associations had standardized effects below 0.1, indicating negligible clinical magnitude consistent with their non-significant p-values.

**Standardized effect sizes for dietary predictor–outcome associations**			
**Outcome**	**Std. B (Ratio)**	**Std. B (Omega-3)**	**Std. B (Omega-6)**
Total Body Fat (%)	-0.016	-0.016	-0.013
Fat Mass (g)	-0.052	0.004	-0.006
Lean Body Mass (%)	0.006	-0.015	-0.001
Lean Mass (g)	-0.048	0.022	0.001
Total Body Mass (kg)	-0.043	0.024	0.002
Body Weight (kg)	0.003	0.016	0.006
BMI (kg/m^2)	0.013	0.017	0.008
RMR (kcal/day)	-0.083	0.075	0.016
RQ	-0.024	0.020	0.043
IL-6 (pg/mL)	0.040	-0.019	-0.004
IL-10 (pg/mL)	-0.031	-0.039	-0.046
CRP (mg/L)	0.049	**0.201**	0.055

Abbreviations: Std. β, standardized beta coefficient; Ratio, dietary omega-6/omega-3 ratio; BMI, body mass index; RMR, resting metabolic rate; RQ, respiratory quotient; IL-6, interleukin-6; IL-10, interleukin-10; CRP, C-reactive protein; SD, standard deviation.
